# Evaluation of the APTIMA Combo 2™ kit for the detection of *Chlamydia trachomatis* and *Neisseria gonorrhoeae* in frozen semen

**DOI:** 10.1007/s10096-025-05138-0

**Published:** 2025-04-30

**Authors:** Thibaut Lutz, Robin Chautard, Maxime Lafontaine, Merve Genc, Arabella Touati, Sabine Pereyre, Olivia Peuchant, Cécile Bébéar, Fabien Garnier, Sébastien Hantz

**Affiliations:** 1 Reproductive Biology Department, CHU Limoges, Limoges, France; 2 Bacteriology Department, National Reference Center for Bacterial STIs, CHU Bordeaux, Bordeaux, France; 3 Bacteriology-Virology-Hygiene Department, CHU Limoges, Limoges, France

**Keywords:** *Chlamydia trachomatis*, *Neisseria gonorrhoeae*, Cryopreserved semen, NAAT

## Abstract

Detection of *Chlamydia trachomatis* (CT) and *Neisseria gonorrhoeae* (NG) in cryopreserved semen is crucial for screening sperm donors. The evaluation of the limit of detection (LOD) of the APTIMA Combo 2™ kit (Panther, Hologic) was performed on cryopreserved semen samples spiked with CT and NG at concentrations ranging from 1 to 106 IFU/mL or CFU/mL, respectively. The LOD was 102 IFU/mL for CT and 10 CFU/mL for NG in single infection or coinfection. An inhibitory effect of semen on amplification was highlighted. This study confirmed the performance of the APTIMA Combo 2™ kit for screening cryopreserved sperm samples before donation.

## Introduction

*Chlamydia trachomatis* (CT) is one of the most prevalent sexually transmitted infections (STI) in the world, with an estimated prevalence in 2020 of 4.0% for women and 2.5% for men aged 15–49 [1]. In men, CT is the second most common cause of urethritis, sometimes complicated by prostatitis or epididymitis [2]. More recently, a meta-analysis has shown that CT can cause infertility in men [3]. In women, the pathology may be asymptomatic or responsible for cervicitis, urethritis, and pelvic inflammatory disease [2] and can also induce infertility and complications during pregnancy [4, 5]. *Neisseria gonorrhoeae* (NG) is a common sexually transmitted infection affecting 82.4 million people worldwide in the 14–49 age group [6]. In men, gonorrhea is symptomatic in 90% of cases, manifesting by an acute urethritis with purulent discharge whereas in women, the infection causes symptoms, such as vaginal discharge, lower abdominal discomfort and dyspareunia, in less than half of cases [7].

In France, good practice rules for medically assisted reproduction (MAR) and gametes donation require molecular testing of sperm donors for CT and NG on a fresh urine sample. However, no recommendations were provided in the context of redirections, where patients who previously benefited of semen cryopreservation in the context of fertility preservation can eventually choose to donate their straws afterwards. In this context, the diagnostic for CT and NG can only be performed “*a posteriori* “on cryopreserved semen.

The nucleic acid amplification test (NAAT) is the reference diagnostic test for the biological diagnosis of CT/NG infections [8, 9]. Early NAATs had a high risk of false negative results when tested in the presence of PCR inhibitors [10, 11]. The Aptima combo 2™ assay (AC2) is a second-generation NAAT that uses target capture, Transcription-Mediated Amplification (TMA™) and Dual Kinetic Assay (DKA) technologies to simplify sample processing, amplify the target rRNA and detect the amplicon, respectively, enhancing sensitivity [12, 13]. While the AC2 kit is FDA-approved for several sample types (endocervical samples, vaginal, anorectal and oropharyngeal swabs and fresh urine), it has not been tested or validated for CT/NG diagnosis in semen. In 2019, the French National Reference Center for Bacterial STIs carried out a limit of detection (LOD) study of the AC2 kit on fresh semen spiked with either CT, NG or *Mycoplasma genitalium* strains. The results showed low LODs for the three bacteria, but highlighted an inhibitory effect when testing for CT and NG in fresh semen with the AC2 kit versus a control medium (2SP) [14].

We sought to address the reproductivity of these results in the cryopreserved semen matrix, as it differs from fresh semen samples through the addition of a cryoprotective medium that usually contains glycerol. Moreover, the previous analysis performed in 2019 did not evaluate the performance of the kit in situations of CT-NG coinfection. The main objective of this study was therefore to evaluate the LODs of the AC2 kit for CT and NG in cryopreserved sperm samples, both separately and in a coinfection situation.

## Materials and methods

Sperm donor straws were used for the experiment. Each donor provided a fresh urine sample to test for CT and NG with the AC2 kit to exclude CT or NG infection. Each semen sample underwent a 0.7 v/v dilution in a cryoprotective medium (SpermFreeze™, Fertipro^®^) following the manufacturer’s recommendations. The cryoprotective solution was a ready-to-use HEPES buffered medium containing physiologic salts, glycine, glucose, lactate and the cryoprotectants glycerol (15%), sucrose (concentration undisclosed) and Human Serum Albumin (4.0 g/L). The resulting mix was stored in CBS™ (Cryo Bio System) high security straws and cryopreserved through slow freezing. These samples are part of the biological collection of the Limoges University Hospital (CRBioLim, AC-2021-4790, certified NF S96-900) authorized by the French Ministry of Health.

The CT reference strain D/UW-3/cx (ATCC^®^ VR-885™, gift from the National Reference Center for Bacterial STIs) and a NG clinical isolate (clinical isolate stored in CRBiolim, the biological resource center of Limoges Hospital) were used for both PBS dilution ranges at concentrations of 1 to 10^6^ IFU or CFU/mL.

First, to determine the minimum test volume required to obtain adequate sensitivity, several volumes were tested: 50, 100 and 150µL. We used the range point to inoculate with CT the glycerol-diluted sperm or control medium (Thermo Scientific UTM™) at concentrations of 1 to 10^6^ IFU/mL. Then to determinate the LOD of CT, NG and CT/NG in the minimal volume, we used the range point to inoculate with CT, NG or both the glycerol-diluted sperm or control medium (Thermo Scientific UTM™) at concentrations of 1 to 10^6^ IFU or CFU/mL. A sample with a given concentration was considered positive if its measured value exceeded the cutoff value in three independent measurements. These different test samples were transferred to Aptima multitest swab transport™ and run in triplicate on the PANTHER ^®^ automated system with Aptima combo 2™ kit.

Means and standard deviations were calculated. Statistics and analysis were carried out with Graphpad.

This study was conducted in accordance with the institution’s procedure (USA-P-015B) for the reuse of health data for research purposes. Remnants of specimens collected for routine clinical care were stored at the Biological Resource Center of the Limoges University (CRBioLim, ISO 20 387 certified). Patients were informed of the possibility of reuse of biological samples for research purposes and could object to this. No personal data from patients were collected for this study and we checked that patients whose samples we used in this study had not objected.

## Results

**Fig. 1:**
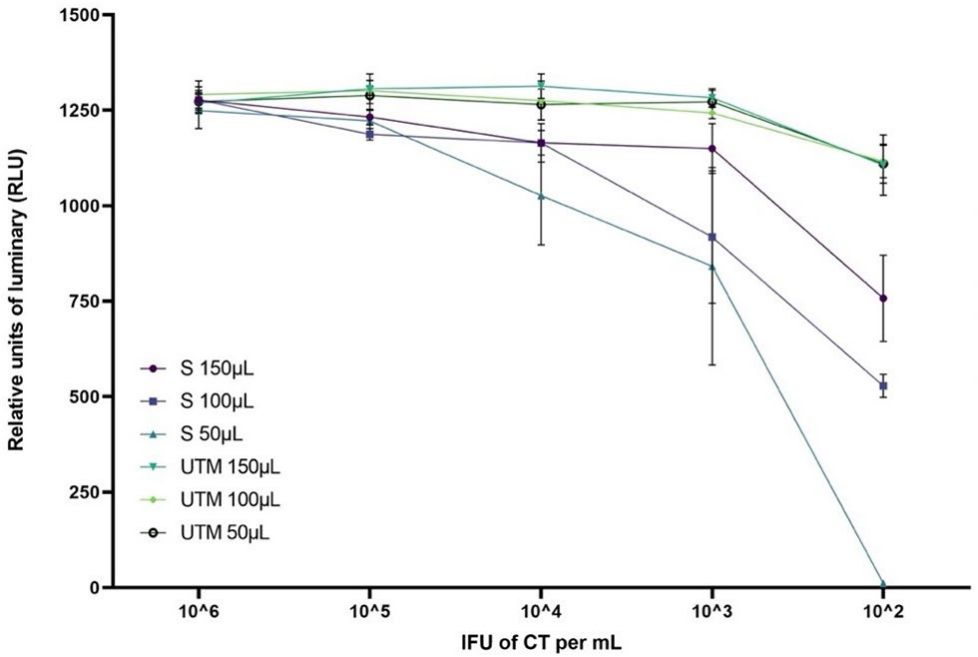
Relative light unit (RLU) measurements in the IFU number per mL of CT in glycerol semen (S) and UTM control (U) at different test portion. Interpretation according to supplier data: CT only, negative if RLU < 25, equivocal for 25 < RLU < 100 and positive if RLU > 100. (Results are expressed in mean and standard deviation from 3 independent experiments)

During the preliminary phase, we studied the inhibitory effect of the matrix and determined the minimum sample volume allowing to maintain the sensitivity of both NAATs. A volume of 150µL sample allowed to obtain the best sensitivity for each of the infectious agents on the different range points, particularly at low concentrations (Fig. 1).

**Fig. 2:**
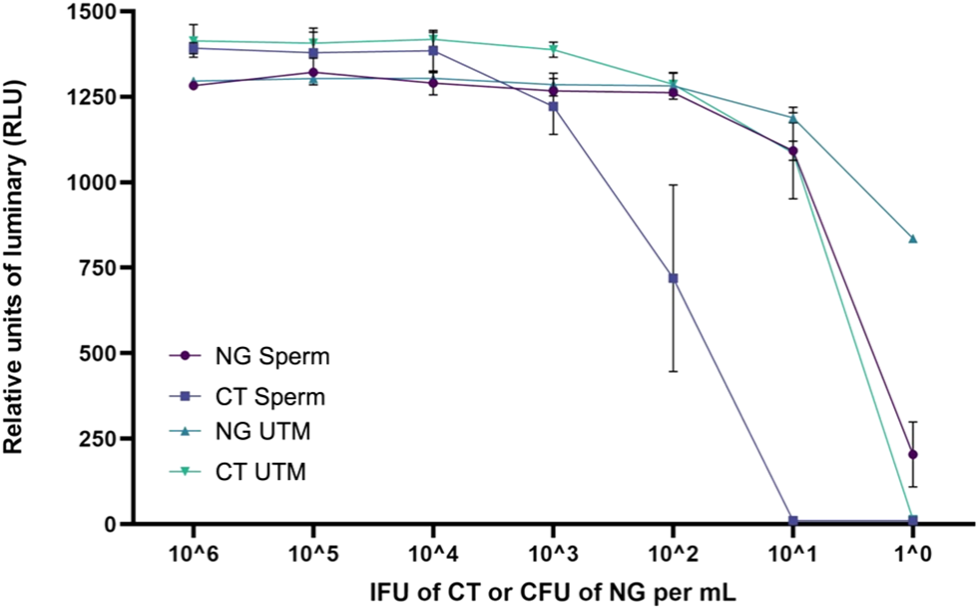
Results of relative light unit (RLU) measurements as a function of bacterial count per mL of NG or CT in glycerol sperm medium (Sperm) and UTM control (UTM), for a 150µL sample. Interpretation according to supplier data: NG only, negative if RLU < 60, equivocal for 60 < RLU < 150 and positive if RLU > 150; NG in co-infection, negative if RLU < 85, equivocal for 85 < RLU < 250 and positive if RLU > 250; CT only, negative if RLU < 25, equivocal for 25 < RLU < 100 and positive if RLU > 100; CT in co-infection, negative if RLU < 85, equivocal for 85 < RLU < 250 and positive if RLU > 250. (Results are expressed in mean and standard deviation from 3 independent experiments)

The LOD of CT and NG for a sample of 150 µL in the frozen sperm/glycerol matrix was estimated at 10^2^ IFU/mL for CT (mono- and co-infection) and 10 CFU/mL for NG (mono- and co-infection). Co-infection had an impact on the LOD of detection in UTM but not in sperm/glycerol matrix. An inhibitory effect of the sperm/glycerol matrix was observed compared with the control UTM medium, with a reduction in sensitivity for the two bacteria tested evaluated at 1 log10 each. This difference was not observed for co-infection (Fig. 2; Table 1).

**Table 1:**
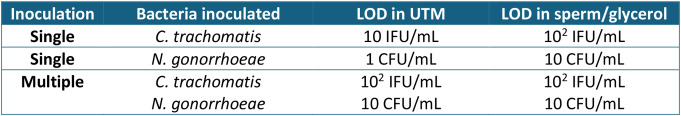
Limit of detection (LOD) results for a 150µl sample, mono- or co-inoculated for *CT* and *NG* in thermo scientific UTM™ control medium and sperm with glycerol medium (70%)

## Discussion

In this study, we evaluated the LOD of the Aptima Combo 2™ kit for CT and NG detection in the frozen sperm matrix and found a LOD of 10^2^ IFU/mL for CT and 10 CFU/mL for NG, both in single and coinfection situations.

These results are consistent with those previously described on fresh semen with the same detection method by the French National Reference Center for Bacterial STIs [14], indicating that the 0.7 v/v dilution in a 15% glycerol cryoprotective medium did not appear to have any additional inhibitory effect on the detection thresholds with Hologic AC2 assay. It confirmed the data described in Hologic’s package insert stating that “vaginal lubricants” were tested for inhibitory interferences and found that “no interference was observed with any of the tested substances” with the AC2 kit. Our results also showed a 1 log difference in LODs between the tested matrix and the control medium, confirming the amplification inhibition effect from semen described in the previous study [14]. Finally, no competitive effect was found in co-infection situations for the detection of both agents, which is an important element as CT and NG association is a frequently encountered clinical situation [14].

While these results are encouraging, several limits must be acknowledged from this work. First, the clinical pertinence of these thresholds has yet to be determined. To our knowledge, very few studies have tried to evaluate the bacterial load of CT and NG in semen of clinically infected patients. In 2022, Dehgan et al. evaluated the mean CT load in fertile vs. infertile patients through a home-made PCR. The mean load was 6.44 log10 copies/ml (range 5.31–7) in infertile men and 4.77 log10 copies/ml (range 4.74–4.8) (*p = 0.044*) in fertile men [15]. Unfortunately, those results cannot be rigorously compared to the LODs described in our studies as the correspondence between IFUs and copies/mL can vary between germs and lab conditions. In 1997, Isbey et al. studied 17 symptomatic patients diagnosed with NG infection and found that the total CFU in semen ranged from 6.9 × 10^4^ to 6.1 × 10^7^ CFU, with an average of 7.0 × 10^6^ CFU [16]. While expressed in a comparable unit, these results were obtained through bacterial culture, which can also vary according to lab practices. Comparison with bacterial loads in other types of samples is also difficult, as the mean CT load is known to vary from a clinical site to another [17]. Of note, the revised 2023 WHO manual for laboratory and point-of-care diagnostic testing for sexually transmitted infections does not include semen in the list of sample types in which screening for CT or NG can be performed.

The reproductivity of these results can also be discussed. Our study involved spiking cryopreserved donor sperm with normal semen parameters. However, clinically infected patients with genito-urinary tract infections can display semen alterations such as leukospermia, volume, pH and viscosity alterations owing to the inflammatory reaction [18]. As we have shown that semen tends to inhibit amplification, confirmation of these results on semen from clinically infected patients could be necessary. Inter-laboratory reproductivity should also be evaluated, as the “frozen semen” matrix depends on lab practices, i.e. the nature of the cryoprotective media used, the dilution volumes, and the freezing and warming procedures.

Defining a relevant detection threshold to ensure the safety of redirected sperm straws is a critical issue in the context of gamete donation. The use of straws containing an inoculum sufficient to infect recipients is cleraly unacceptable from a patient safety perspective, as CT and NG can cause genital infections with serious consequences, particularly impaired fertility in women. Furthermore, while the direct impact of CT and NG sperm parameters (motility, vitality and DNA fragmentation) remains debated in the literature [19, 20], their association with male infertility seems to be strongly suspected [3, 21, 22].

Therefore, the use of contaminated straws could not only present a risk for recipients’ health but might also reduce their chances of success in assisted reproductive treatments involving redirected sperm straws. In that regard, systematic screening for CT and NG in validated matrixes such as urine samples before fertility preservation could represent an efficient preemptive measure, provided informed patients’ consent is obtained.

In conclusion, our study confirms interesting detection thresholds of the AC2 kit for CT and NG detection. It shows any difference in inhibitory effect between the frozen sperm matrix and the previously described fresh sperm matrix. Clinical data on mean bacterial loads of CT and NG in semen remain necessary to confirm the pertinence of these LODs, so as to clear redirection straws for sperm donation without any risk of transmission of CT or NG to recipients.

## Data Availability

No datasets were generated or analysed during the current study.
